# ICTV Virus Taxonomy Profile: *Finnlakeviridae*

**DOI:** 10.1099/jgv.0.001488

**Published:** 2020-08-25

**Authors:** Sari Mäntynen, Elina Laanto, Lotta-Riina Sundberg, Minna M. Poranen, Hanna M. Oksanen, ICTV Report Consortium

**Affiliations:** ^1^​ Molecular and Integrative Biosciences Research Programme, Faculty of Biological and Environmental Sciences, University of Helsinki, Viikinkaari 9, 00014 University of Helsinki, Finland; ^2^​ Department of Microbiology and Molecular Genetics, University of California, Davis, One Shields Avenue, Davis, California 95616, USA; ^3^​ Department of Biological and Environmental Science and Nanoscience Center, University of Jyväskylä, Survontie 9, 40014 University of Jyväskylä, Finland

**Keywords:** ICTV report, taxonomy, *Finnlakeviridae*, Flavobacterium phage FLiP, icosahedral membrane-containing virus, single-stranded DNA phage

## Abstract

*Finnlakeviridae* is a family of icosahedral, internal membrane-containing bacterial viruses with circular, single-stranded DNA genomes. The family includes the genus, *Finnlakevirus*, with the species, *Flavobacterium virus FLiP*. Flavobacterium phage FLiP was isolated with its Gram-negative host bacterium from a boreal freshwater habitat in Central Finland in 2010. It is the first described single-stranded DNA virus with an internal membrane and shares minimal sequence similarity with other known viruses. The virion organization (pseudo *T*=21 *dextro*) and major capsid protein fold (double-β-barrel) resemble those of Pseudoalteromonas phage PM2 (family *Corticoviridae*), which has a double-stranded DNA genome. A similar major capsid protein fold is also found in other double-stranded DNA viruses in the kingdom *Bamfordvirae*. This is a summary of the International Committee on Taxonomy of Viruses (ICTV) report on the family *Finnlakeviridae*, which is available at ictv.global/report/finnlakeviridae.

## Virion

**Fig. 1.**
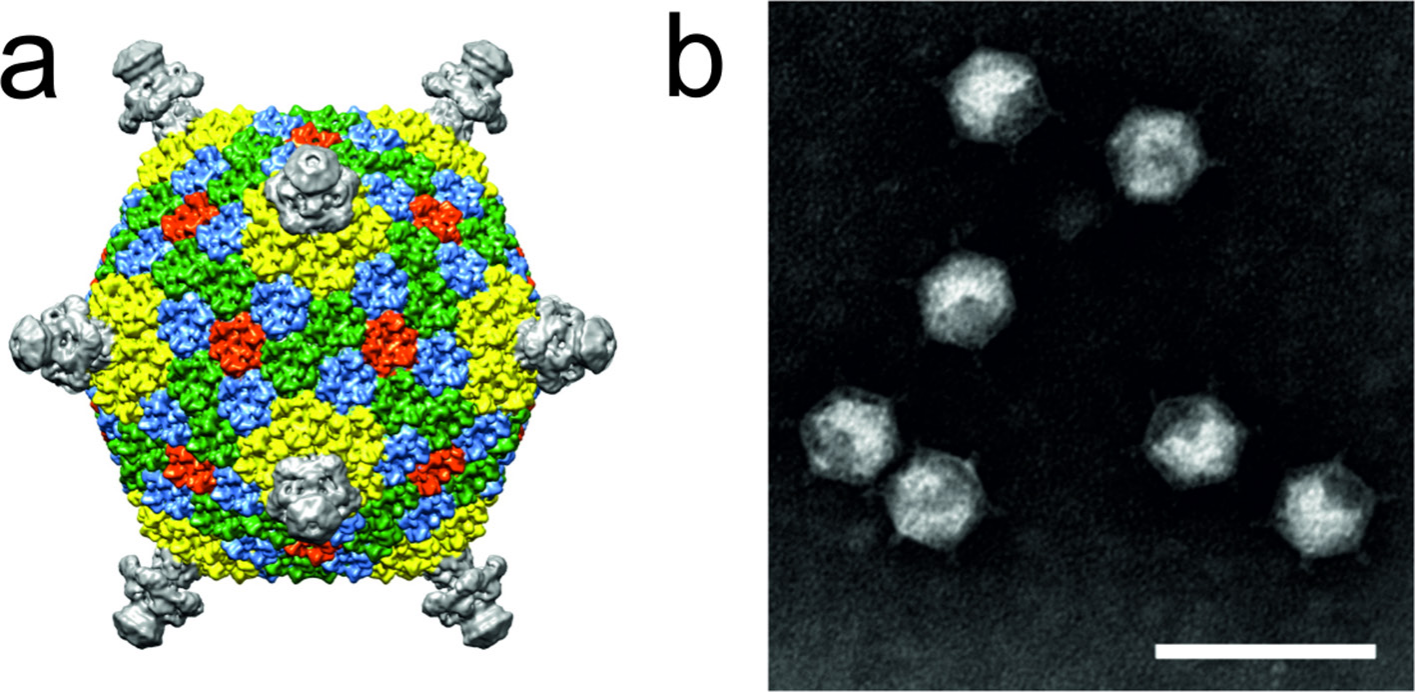
Flavobacterium phage FLiP virion structure. (a) Cryo-electron microscopic reconstruction [1]. (b) virions negatively-stained with 2 % phosphotungstic acid (pH 8.5) and visualized under transmission electron microscopy. Scale bar represents 100 nm.

The virion of Flavobacterium phage FLiP consists of an icosahedral protein shell and an internal membrane, uniquely combined with a circular ssDNA genome [1] (Table 1, Fig. 1). The diameter of the virion is about 59 nm (vertex-to-vertex). Pentameric spike complexes protrude about 12 nm from the protein shell surface at the fivefold vertices. The inner surface of the icosahedral protein capsid is covered by a lipid bilayer membrane (5 nm thick), enclosing the single-stranded DNA genome. The major capsid proteins (MCPs) forming the outer protein shell follow a pseudo *T*=21 *dextro* icosahedral capsid organization, previously described only for the marine double-stranded DNA Pseudoalteromonas phage PM2 [2]. The Flavobacterium phage FLiP MCPs consist of two β-barrels with jellyroll topology and arrange into trimers to form pseudohexameric molecules [1]. A similar major capsid protein fold has also been described for members of the kingdom *Bamfordvirae* [3], such as Enterobacteria phage PRD1 (family *Tectiviridae*) [4].

**Table 1.** Characteristics of members of the family *Finnlakeviridae*

Typical member:	Flavobacterium phage FLiP (MF361639), species *Flavobacterium virus FLiP*, genus *Finnlakevirus*
Virion	Icosahedral, internal membrane-containing virions, approximately 59 nm in diameter. Spikes protrude from the virion surface
Genome	9.2 kb of circular, single-stranded DNA
Replication	Possibly rolling circle replication
Translation	By the host translation machinery
Host range	Gram-negative bacteria from the genus *Flavobacterium *
Taxonomy	The genus *Finnlakevirus* includes the species *Flavobacterium virus FLiP*

## Genome

**Fig. 2.**
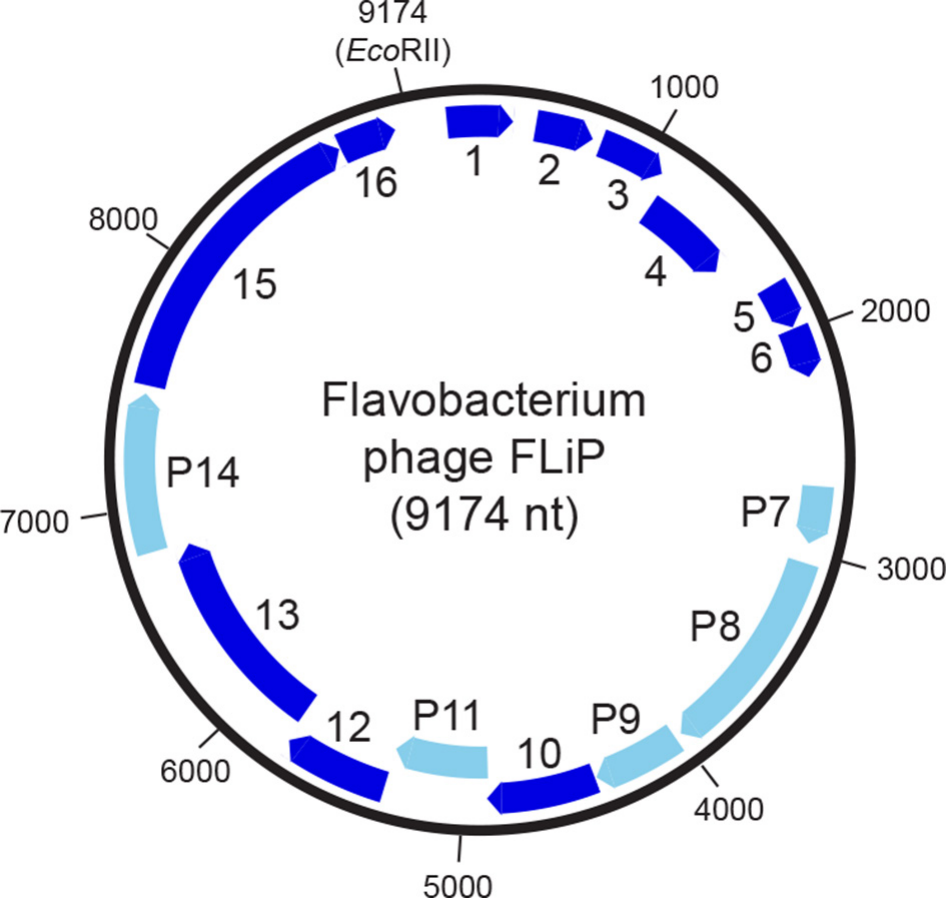
Genome organization of Flavobacterium phage FLiP. Arrows show the direction of transcription of predicted CDSs, with light blue indicating those encoding the structural proteins P7–9, P11 and P14. The unique *Eco*RII restriction site is at nucleotide position 1.

Virions contain a single copy of a circular single-stranded DNA of 9174 nucleotides with a GC content of 34 % [1]. 16 predicted coding sequences (CDS), all in the same orientation (Fig. 2), show limited similarity with other known sequences. Five CDSs have been shown to encode structural proteins (CDS7–9, 11, 14). There are sequence similarities between CDS14 and several lytic transglycosylases and between CDS15 and rolling circle replication proteins [1, 5].

## Replication

Flavobacterium phage FLiP is a virulent virus that induces host-cell lysis at the end of the viral reproduction cycle [1]. CDS14 is predicted to encode a virion-associated lytic protein likely assisting in the penetration of the host peptidoglycan layer during entry. Flavobacterium phage FLiP does not encode any identifiable DNA or RNA polymerase. However, the sequence resemblance of CDS15 to rolling circle replication proteins suggests a replication mechanism typical of single-stranded DNA viruses [1, 5]. The absence of a putative packaging ATPase implies that a viral-encoded ATPase is not required for the encapsidation of the genome [5].

## Taxonomy

Current taxonomy: ictv.global/report/finnlakeviridae. The family *Finnlakeviridae* contains the genus *Finnlakevirus* with the species *Flavobacterium virus FLiP*. Based on phylogenetic analysis of MCP sequences, Flavobacterium phage FLiP forms its own group among the tailless prokaryotic DNA viruses and proviruses with double-β-barrel MCPs [5].

## Resources

Current ICTV Report on the family *Finnlakeviridae*: ictv.global/report/finnlakeviridae
